# Comparison of linear distance of glenoid fossa to frontomaxillary nasal suture in skeletal Class-II and skeletal Class-I malocclusion

**DOI:** 10.12669/pjms.40.7.8506

**Published:** 2024-08

**Authors:** Sadaf Siddiqui, Ambreen Afzal Ehsan, Hasnain Sakrani, Sadia Asif Samdani

**Affiliations:** 1 Dr. Sadaf Siddiqui, BDS FCPS (Orthodontics), CHPE Senior Registrar, Department of Orthodontics, Altamash Institute of Dental Medicine, Karachi, Pakistan; 2 Prof. Dr. Ambreen Afzal Ehsan. BDS, FCPS (Orthodontics) C. Ortho, F-TMJ, OFSOS, M. Orth Head of Orthodontics Department & Dean of Academics, Department of Orthodontics, Altamash Institute of Dental Medicine, Karachi, Pakistan; 3 Prof. Dr. Hasnain Sakrani, BDS FCPS. Principal, Department of Orthodontics, Altamash Institute of Dental Medicine, Karachi, Pakistan; 4 Dr. Sadia Asif Samdani, BDS FCPS (Orthodontics) Assistant Professor, ISRA Dental University, Hyderabad, Pakistan. Department of Orthodontics, Altamash Institute of Dental Medicine, Karachi, Pakistan

**Keywords:** Fronto maxillary nasal suture, Glenoid fossa, Mandibular retrusion Skeletal Class-II Skeletal Class-I

## Abstract

**Objective:**

To compare linear distance of glenoid fossa to frontomaxillary nasal suture in skeletal Class-I and II malocclusions.

**Methods:**

This cross-sectional study was conducted at the Department of Orthodontics, Altamash Institute of Dental Medicine, Karachi Pakistan. The duration of study was one year from January, 2019 to January, 2020. After taking informed consent from patient and hospital ethical committee a total of 60 patients were included in the study using WHO sample size calculator. Two groups comprising 30 patients each i.e., Skeletal Class-I malocclusion and Skeletal Class-II malocclusion with mandibular retrusion both having normal vertical relationship were included in the study. The cephalometric measurements SNA, SNB, SNMP, FHMP, GF-FMN, CO-GO, CO GN on lateral cephalograms were measured and compared between the two groups. Independent t test was applied and p value ≤ 0.05 was considered as significant.

**Results:**

In skeletal Class-I malocclusion the mean linear distance of GF-FMN was 70.2 ± 4.02 mm and in skeletal Class-II malocclusion it was 73.4 ± 4.04 mm (p value .004). Glenoid fossa was 3.2 mm distally placed in patients with Class-II malocclusion.

**Conclusion:**

Glenoid fossa position is a diagnostic feature of Class-II malocclusion associated with mandibular retrusion. One of the effective cephalometric measurements to check glenoid fossa position is the distance from the glenoid fossa(GF) to the frontomaxillary nasal suture FMN (GF-FMN).

## INTRODUCTION

Craniofacial bones of skull and jaw should be proportionate as disproportion would lead to sagittal and vertical discrepancies of jaws.^1^ The cranial base has anterior and posterior parts, in which maxilla is directly attached to the anterior region through sutures and the mandible is indirectly attached to the posterior part through the temporomandibular joint. Temporomandibular joint (TMJ) connects jawbone to the skull. It is attached to the temporal bone of the skull above and the mandible below. Since the TMJ is connected to the mandible, the right and left joints must function together and therefore are not independent of each other Therefore, any change in the amount or direction of growth of the cranial base can have a direct effect on maxilla or indirect effect on the mandible. Skeletal discrepancies of vertical and anteroposterior direction are dependent on association of maxilla and mandible to cranium.^2^ The position of glenoid fossa which is the temporal part of temporomandibular joint where the mandible positions itself determines the craniofacial appearance. Its distal placement leads to mandibular retrusion^3^ whereas forward placement of the joint causes mandibular protrusion.^4^ Treatment modality changes when mandible is retrusive and glenoid fossa is distally placed. There is limited data about the significance of the position of the glenoid fossa of temporomandibular joint. Studies show significant changes that can be induced in the structural features of the posterior wall of the glenoid fossa following mandibular advancement.^5^

Bjork A et al. was the first to demonstrate significant relation of cranial base and jaw.^6^ Brain is supported by cranial base. During growth, articulation of neurocranium, viscerocranium and mandible with base of cranium leads to different movements of maxilla and mandible. This leads to a change in glenoid fossa and condylar position.^2^

The cranial base has a direct relationship with the anteroposterior position of jaws.^7^ Anatomy of the craniofacial region can affect the position and size of the condyle and glenoid fossa.^8^ There is a difference in the antero-posterior location of glenoid fossa between Class-II and Class-III malocclusion.^9^

More studies are needed to establish reference values for measurements in Pakistani population involving glenoid fossa in different ages and with different dento skeletal relationships.^10^ Present study aimed to compare linear distance of glenoid fossa in skeletal Class-I and II malocclusions. Induction of structural changes in posterior wall of glenoid fossa can be a viable treatment option in 10-12 years old Class-II patients with distally placed glenoid fossa. It is the first of its kind where these relationships are studied in pediatric population. This would benefit Orthodontist to intervene and plan treatment.

## METHODS

This cross-sectional study was conducted at the Department of Orthodontics, Altamash Institute of Dental Medicine, Karachi Pakistan. The study was conducted in Orthodontics department, Altamash Institute of Dental Medicine, Karachi Pakistan from January, 2019 to January, 2020 for 12 months. Patients informed consent was taken for participate in study. WHO sample size calculator was used, taking statistics 67.6 ± 35 for margin of error = 0.9, the calculated sample size was 60.2. Two groups comprising of 30 patients each of 10-12 years i.e., Skeletal Class-II malocclusion (ANB angle>4^0^) with mandibular retrusion angle SNB (<76^0^<70^0^) and skeletal Class-I malocclusion, (ANB angle 0-4^0^). Both having normal vertical relationship SNMP (28^0^<37^0^) were included in the study. The cephalometric measurements on lateral cephalograms of linear distance of glenoid fossa to frontomaxillary nasal suture were measured and compared between the two groups. Independent t-test was applied and p-value ≤ 0.05 was considered as significant. Patients with tooth agenesis, supernumeraries; dentofacial trauma, temporomandibular joint abnormalities, complex genetic syndromes and previous orthodontic treatments were excluded.

Patients’ pretreatment cephalometric radiograph was taken for record. Lateral cephalograms of patients meeting inclusion criteria were traced manually on acetate sheets with a 0.5 mm mechanical lead pencil in a dark room with conservative methods and linear and angular measurements were recorded. The linear distances as glenoid fossa (GF) to fronto maxillary nasal suture (GF-FMN), Mandibular length from Condylion to Gnathion (Co-Gn), ramal length from Condylion to Gonion (Co-Go), in both groups were measured. Angular measurements of Sella Nasion with point A(SNA), Sella Nasion to point B(SNB), difference between SNA and SNB (ANB) Sella Nasion to Mandibular plane(SNMP) were recorded.

**Fig.1:**
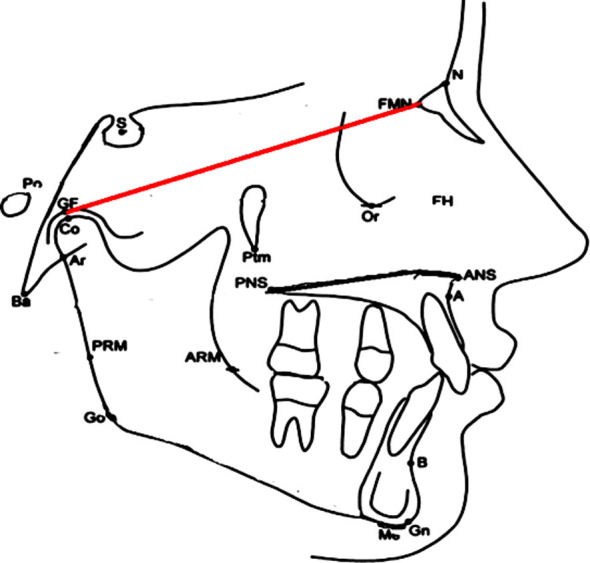
GF; Glenoid fossa; FMN (Fronto-Maxillary Nasal suture).

**Fig.2:**
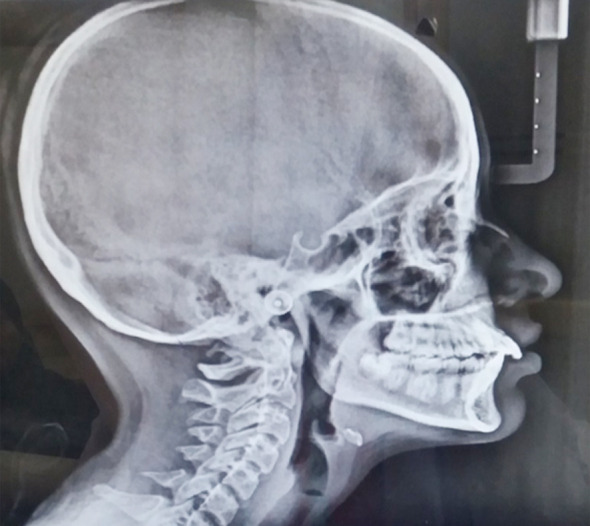
Lateral cephalogram of patient in skeletal Class-II malocclusion.

**Table-I:** Mean and Standard Deviation of Age and cephalometric variables in male and female patients

Parameters	Overall (n=60)	Male (n=24)	Female (n=36)
***Age in years***			
10	8 (13.3%)	4 (16.7%)	4 (11.1%)
11	4 (6.7%)	4 (16.7%)	-
12	48 (80.0%)	16 (66.7%)	32 (88.9%)
Range	10-12	10-12	10-12
Mean ± S.D	11.7 ± 0.71	11.5 ± 0.78	11.8 ± 0.64
***Skeletal Class***			
I	30 (50.0%)	12 (50.0%)	18 (50.0%)
II	30 (50.0%)	12 (50.0%)	18 (50.0%)
***GF- FMN length***			
Range	65 -77	66 - 77	65 – 77
Mean ± S.D	71.8 ± 4.29	73.0 ± 3.47	70.9 ± 4.63
***Angle SNA***			
Range	77 – 84	77 – 84	77 – 84
Mean ± S.D	80.7 ± 1.92	80.6 ± 2.30	80.7 ± 1.66
***Angle SNB***			
Range	71 – 79	71 – 79	75 – 79
Mean ± S.D	75.9 ± 1.89	75.2 ± 2.33	76.5 ± 1.32
***Angle ANB***			
Range	1 – 9	2 – 9	7 – 9
Mean ± S.D	4.5 ± 1.99	5.0 ± 1.69	4.2 ± 2.14
***Angle SNMP***			
Range	23 – 42	26 -66	23 – 42
Mean ± S.D	32.3 ± 4.60	32.0 ± 3.54	32.5 ± 5.23
***Angle FHMP***			
Range	19 – 37	21 – 31	19 -37
Mean ± S.D	25.6 ± 4.58	25.7 ± 3.32	25.5 ± 5.31
***COGN length***			
Range	85 – 113	93 – 107	85 – 113
Mean ± S.D	101.1 ± 4.68	101.9 ± 4.18	100.5 ± 4.95
***COGO length***			
Range	41 – 58	43 – 58	41 – 56
Mean ± S.D	51.7 ± 4.52	53.3 ± 4.72	50.7 ± 4.11

### Ethical Approval

The Research Ethics Committee of Institute approved research protocol (ERB no AIDM/REC/6/2020/01).

Demographic variables such as patients age and gender was recorded. Data was analyzed by SPSS21. The mean and standard deviations (Sd) of age, SNA, SNB, ANB, Co-Gn, Co-Go, point GF, GF-FMN, GF and FMN, SNMP were calculated. Frequency and percentage were calculated for gender. Two groups were compared by independent t-test for linear distances p-value ≤ 0.05 was taken as significant. Effect modifiers like age, gender was addressed through stratification, post stratification independent t- test was carried out. The comparisons between the Class-II group and skeletal Class-I group on the cephalometric measurements for the assessment of glenoid fossa position was done by independent t-test and p value ≤ 0.05 was taken as significant.

## RESULTS

Total of 60 patients participated in the study. There were 24(40%) males and 36 (60%) females. Mean ± SD of age in skeletal Class-I, 11.5 ± 0.90 was less as compared to skeletal Class-II, mean±SD 11.9 ± 0.35 (p-value = 0.027). The mean ± SD of angle SNA was 80.2 ± 1.72 in skeletal Class-I and in skeletal Class-II 81.2 ± 2.02 (p-value=0.044). In skeletal Class-I the linear distance of GF to FMN suture was 70.2 ± 4.02 mm and in Class-II it was 73.4 ± 4.04 mm (p value .004). Glenoid fossa was 3.2 mm distally placed in Class-II.

**Table-II:** Comparison of Mean and Standard deviation of age and cephalometric variables between Skeletal Class-I and II

Variables	Skeletal Class I (n=30)	Skeletal Class-II (n=30)	P-value

	Mean ± S.D	Mean ± S.D	
Age in years	11.5 ± 0.90	11.9 ± 0.35	0.027
GF-FMN length	70. 2 ± 4.02	73.4 ± 4.04	0.004
Angle SNA	80.2 ± 1.72	81.2 ± 2.02	0.044
Angle SNB	77.1 ± 1.28	74.8 ± 1.65	0.001
Angle ANB	2.9 ± 1.27	6.2 ± 0.92	0.001
Angle SNMP	32.4 ± 4.34	32.1 ± 4.91	0.803
Angle FHMP	25.6 ± 3.52	25.5 ± 5.51	0.978
COGN length	102.1 ± 4.43	100.1 ± 4.79	0.110
COGO length	53.4 ± 3.58	50.1 ± 4.78	0.003

## DISCUSSION

Class-II craniofacial defects in primary dentition can settle in growth phase however, these differences may not be corrected due to difference in degree and pattern of growth in Class-II and Class-I malocclusion individuals.^11^ Anatomical features of condyle and glenoid fossa differ in patients of different sagittal skeletal patterns; therefore, it should be given importance before treatment planning.^10^

It is crucial to appreciate an area on craniofacial complex as cause of malocclusion and needs to be addressed. Importance of glenoid fossa must be acknowledged in shaping craniofacial anatomy of skeletal Class-I, Class-II and GF location must be looked before making the treatment plan of these skeletal malocclusions.^12^

In our study 36(60%) girls and 24(40%) boys were present. This difference of gender in our study was because of small sample size, more adolescent female patients under treatment between age group of 10-12 years. In a study done by de Mattos JM et al males and females in the sample was similar in all groups.^13^

We have taken the distance from glenoid fossa to frontomaxillary nasal suture. This is because GF-FMN has a significant relation with the angulation between the posterior and anterior portions of the cranial base.^14^ Mushtaq M et al.^15^ did a study in which mean of glenoid fossa to frontomaxillary nasal suture (GF-FMN) distance in skeletal Class-II group was 80.50+6.17 while it was 77.72+7.69 in skeletal Class-I group. In their study Glenoid Fossa-Sella (GF-S) glenoid fossa position with sella on frankfort horizontal plane in both groups was 17.47+3.45 and 17.01+3.79, but it was statistically not significant (p < 0.528). GF and FMN distance in analysis of skeletal malocclusions of Class-II and Class-I is better than GF-S relation.

Many studies in the past have suggested that a large cranial base angle is the cause of skeletal Class-II pattern with a distal position of the temporomandibular joint within the skull^1^ but the length GF-FMN provides an easy and effective measure to check the cause of malocclusion. That is why in our study only GF-FMN distance was measured.

In our study only normal skeletal vertical relationship cases were included. Miranda et al.^16^ found that vertical distance between the fossa and condyle has an effect on the position of fossa as the condyle was more superiorly positioned in patients with high vertical pattern as compared to low vertical patterns.

In skeletal Class-I the distance of glenoid fossa to frontomaxillary nasal suture (GF-FMN suture) was about 70.2 ± 4.02 mm and in Class-II it was 73.4 ± 4.04 mm which confirms that glenoid fossa is 3.2 mm distally placed in Class-II. Giutini V^3^ conducted the study in which the average distance from the glenoid fossa to frontomaxillary nasal suture in the Class II group was 3.5 mm longer than the average distance in the control group. Mushtaq M et al.^15^ also showed in their study that the glenoid fossa is located significantly posteriorly in skeletal Class-II than in Class-I (p < 0.05).

By only taking Class-II malocclusion cases of mandibular retrusion, the study was able to pinpoint the exact cause of Class-II malocclusion. This result is statistically and clinically important, as it is evident that in the absence of any other dentofacial problems such as mandibular size deficiency or any vertical problems, Class-II malocclusion in the individual patient can be related to a distal position of the glenoid fossa resulting in substantial mandibular retrusion.

Angle SNA showed that cases with maxillary protrusion were excluded and angle SNB showed the presence of mandibular retrusion. The increases in ANB angle values confirmed that there was an increase due to patients taken with mandibular retrusion. The mean value of SNB was 74.8^0^ in Class-II group. In previous studies it was established by the values for SNA, SNB and ANB that cranial base has an effect on maxillary growth.^17^ Mandibular parameters like Co-Gn, Angle SNMP and angle FHMP did not reveal statistically significant differences between the two groups.

Indicators of mandible such as length of ramus measured from most superior part of condyle (Condylion Co) to angle of mandible (Gonion Gn) did not reveal statistically significant differences between the two groups. In previous studies there were differences found between ramal length i.e from condylion to gonion or ramus width i.e gonion to menton but in our study we did not find significant differences in length between the two groups.^2^ Although the length of mandibular body measured from superior part of condyle (condylion Co) to mid point of chin (Gnathion Gn) showed significant difference in Class-II group being shorter than Class-I. It is now emphasized that the distal position of the glenoid fossa, should be considered in treatment planning as a condition which predisposes to Class-II malocclusion leading to posteriorly positioned mandible.

From this study we conclude that orthopedic effect of functional appliances as removable functional appliances Clarks twin block activator or fixed functional as Herbst, MARA and many others can be given to induce growth in the posterior wall of the glenoid fossa to advance the mandible and mechanically stimulate condylar growth. Nindra et al.^18^ Sharma V et al.^19^ have conducted many studies of functional appliances orthopedic effects on glenoid fossa position. These changes can contribute significantly to the correction of Class-II malocclusion associated with mandibular retrusion.

### Limitations

We did not use digital cephalograms to avoid human error. Ethnicity and local demographic were not taken into consideration and further longitudinal studies may be done to document growth modifications effect on linear distance.

## CONCLUSION

A major diagnostic feature of Class-II malocclusion due to posteriorly positioned mandible is posterior position of the glenoid fossa. The distance of glenoid fossa to frontomaxillary nasal suture GF-FMN is an effective measurement to evaluate glenoid fossa position.

### Authors’ Contributions:

**SS**: conceived, designed, statistical analysis, editing of manuscript and responsible for the accuracy of the study.

**AA and HS**: Supervised, reviewed and did final approval of manuscript.

**SA**: did data collection and manuscript writing.
